# Localized Malignant Myxoid Anaplastic Mesothelioma of the Pericardium

**DOI:** 10.4021/jocmr2009.05.1241

**Published:** 2009-06-21

**Authors:** Guang Zhi Yang, Jing Li, Hua Ye Ding

**Affiliations:** a Department of Pathology, the General Hospital of Beijing Military Command, Beijing, 100700, China

## Abstract

Primary malignant mesothelioma of the pericardium is a rare cardiac neoplasm. Most are diffuse and have poor prognosis with median survival of six months. In the paper, we describe a young male patient having no exposure history of asbestos with localized malignant myxoid mesothelioma of the pericardium. The tumor displayed significant myxoid change in stroma, and anaplastic cytology, including pleomorphy, poor cohesion, prominent nuclei, with high mitoses, which led to difficulty in diagnosis. The tumor showed typical immunohistochemical phenotypes of mesothelioma, positive for WT-1, calretinin and CK5/6. Ki-67 labeling index was about 50% in general and nearly 80% in the most active areas. The patient showed better outcome. The report suggests the diagnosis of myxoid mesothelioma is supposed to rely on clinical data, and immunohistochemistry is assumed to be for differentiation.

**Keywords:**

Myxoid mesothelioma; Anaplastic; Diagnosis; Immunohistochemistry; Prognosis

## Introduction

Primary malignant mesothelioma of the pericardium is a rare cardiac neoplasm, accounting for approximately 0.0017% of cardiac and pericardial tumors [1]. As far as mesothelioma is concerned, pericardial mesothelioma represents no more than 1% of all [2]. In macroscopy, most fill the pericardial cavity or spread diffusely over the surface. Histologically, mesothelioma is classified into four main subtypes, which are epithelioid, sarcomatoid, desmoplastic and biphasic. However, it is recognized of a striking variation in the morphology. For example, myxoid change is an infrequent phenomenon taking place in epithelioid mesothelioma. Furthermore, it may be so obvious that cases of which at least 50% area containing a pronounced myxoid stroma that occupied more than 50% of the tumor volume are designated as myxoid mesothelioma [3]. Since myxoid mesothelioma, as one sort of seldom seen mesothelioma, was first described by Butchart in 1976[4], several reports in small series have presented, but none of the pericardium was seen. In most reported cases, the tumor cells were remarkably bland. In the paper, we reported one case of myxoid mesothelioma of the pericardium, forming a bulky neoplasm in the mediastinum with anaplastic cytologic characteristics.

## Case Report

A 23-year-old male presented with growing dyspnea in recent six months, who denied history of smoking and exposure to asbestos. CT scans demonstrated a pleural effusion and a well demarcated tumor in mediastinum. At surgery the neoplasm was found to occupy most of the anterior mediastinum and grew on a slim pedicel connected with the pericardium.

Gross investigation revealed an ovoid mass measured 9 cm × 5 cm × 4 cm with a smooth intact capsule and myxoid external appearance. The cut surface was obviously myxoid change with haemorrhage and necrosis.

**Figure 1:**
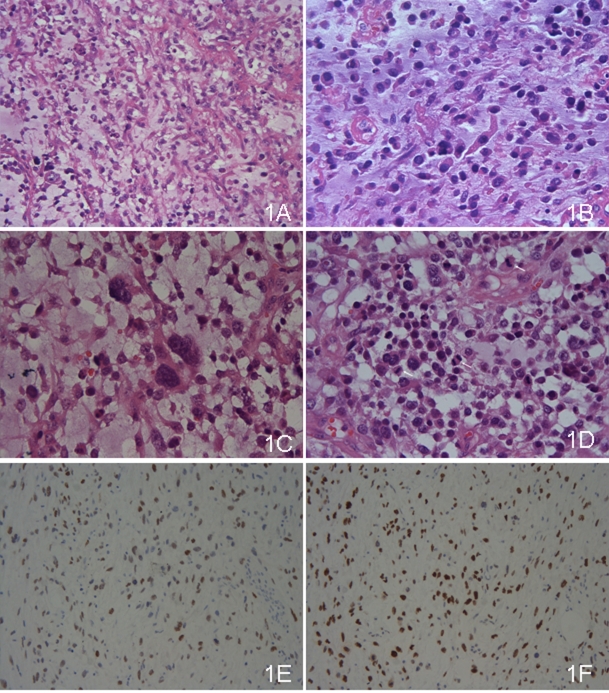
(A) The tumor was composed of abundant myxoid stroma comparted with fibrous tissues and veins, in which the tumor cells floated. Most of the tumor cells were medium, whereas some were large. The cells were lack of cohesion (×100, HE). (B) The tumor cells had ample amount of eosinophilic cytoplasms with coarse and prominent nuclei. The cytoplasms were irregular, some like the red streamer (×400, HE). (C) The giant odd cells were present (×400, HE). (D) Mitosis figures were common, and three were marked in the high power field (×400, HE). (E) WT-1 was positive. (F) Ki-67 labeling index was high.

The tumor was composed of abundant myxoid stroma comparted with fibrous tissues and veins, which was easily recognized even in low power scanning. The tumor cells, floated in such mucin pools, admixed with inflammatory cells (Fig. 1A). Great variances were present in cell density and size (Fig. 1B). The compact areas were composed mainly of tumor cells of medium size. Alternately, the larger cells were inclined to assemble in the loose areas, where giant odd cells were also occasional (Fig. 1C). The cells lacked cohesion, nearly individually dispersed, which were more significant in large cells. The tumor cells were epithelioid with eosinophilic and irregular polygonal cytoplasms. The amounts and figures of cytoplasm were diverse, from a few to much, some even like the red streamer. The nuclei were coarse and prominent, whereas nucleoli were not common. Mitosis figures were quite frequent and estimated of more than 10 per 10 high power fields (Fig. 1D).

Immunohistochemical study displayed that the tumor cells were strongly positive for WT-1 (Fig. 1E), moderately positive for calretinin and CK5/6, weakly positive for AE1/AE3, and locally positive for vimentin. Other immunostaining items, including EMA, CEA, CgA, Syn, actin(pan), Desmin, SMA, myoD1, CD34, CD31, HMB45, Melan A, S-100, CD30, bcl-2 and CD99, were all negative. The background cells were positive for LCA and CD68, which were regarded as lymphocytes and monocytes. The tumor cells were also diffusely positive for P53. The labeling index of Ki-67 staining was about 50% in general and nearly 80% in the most active areas (Fig. 1F).

## Discussion

Primary malignant mesothelioma of the pericardium is a rare cardiac lesion, which is diagnosed when such is excluded outside the pericardium. The pericardial mesothelioma occurs in a wide range of ages with mean of 45, just like in other sites [1]. It is believed that a large proportion is induced by asbestos. Most displays pericardial thickening or diffusely spreading over the pericardium. Our patient was young without exposure history of asbestos, and showed a localized lump in the mediastinum without thickening of the pericardium or pleura. Clinically, the patient mainly displayed dyspnea, just like the general. However, the underlying reason was assumed of extrusion to the lungs from the tumor rather than effusions, even the patient also had a little.

The main challenge of the report was what to diagnose, which verified that mesothelioma has so many variants in histological morphology leading to difficulty in diagnosis sometimes. The case was effluent in myxoid stroma and anaplastic tumor cells, which brought up with no explicit consideration clue. In our diagnosis experience, nearly all soft tissue tumors which could happen in mediastinum were excluded by immunohistochemistry, such as solitary fibrous tumor, rhabdomyosarcoma, synovial sarcoma, leiomyomatous tumors, neurogenic tumors, and melanoma, lymphoma, even anaplastic carcinoma. The case displayed a localized mass, which was seldom present in mesotheliomas, but the important information that the tumor had a stalk connected with the paracardiac offered by the surgeons, sparked possibility of mesotheliomas. And the immunostaining results displayed that WT-1, calretinin and CK5/6 were positive, then diagnosis of mesothelioma was confirmed [5].

Until now, descriptions about myxoid mesothelioma were in small series [6, 7]. In a large review, Shia et al demonstrated mxoid mesotheliomas represented 8% of their series of 234 cases [3]. They also identified that myxoid stroma was mainly composed of hyaluronidase-sensitive material. To recall normal mesothelial cells have the function of secreting glycosaminoglycans, particularly hyaluronic acid, it is easily understood that myxoid mesothelioma retained the function of the normal counterparts.

To the note, there are another group of mesotheliomas, which called mucin-positive mesothelioma [8]. The mucin was also hyaluronic acid type, however, it was confined to the cells, rather than extracellular myxoid stroma. Such group also displayed aberrant immunostaining for markers of adenocarcinoma such as CEA, which was not seen in myxoid mesotheliomas. It is supposed that the two mucin-rich mesotheliomas were not the same group of mesotheliomas.

Overall, the prognosis of the pericardial mesothelioma remains very poor with median survival of about six months after diagnosis and treatments such as surgery, radiotherapy and chemotherapy have modest efficacy. The literature has all prompted that myxoid mesothelioma patients have a better prognosis than patients with other subtypes, which was reinforced by the morphology and immunohistochimestry. The tumor cells of myxoid mesothelia were always epithelioid without significant atypia, and mitoses were not comman. Ki-67 index was testified to suggest survival in patients with mesotheliomas [9]. In the past reported cases, the Ki-67 index was either minimal (less than 5%) or moderate (less than 30%), which displayed a low proliferation rate in general. Contrarily, our case was quite different. The tumor cells are anaplastic, and mitoses were also common, estimated more than 10 per 10 high power fields. The Ki-67 labeling index was high, and nearly 80% in the most active areas. It was also thought to reach the diagnosis standard of pleomorphic mesothelioma. All these implied the poor prognosis. To make it more complicated, the case was the localized type, which was figured of more favorable outcome than the diffuse type.

The plus and minus factors admixed, which made it more difficult to judge the prognosis. The case still survived one year after surgery without further radiotherapy and chemotherapy.

According to our experiences, the mesotheliomas are very heterogeneous in morphology. The clinical features, including exact tumor positions and connections with circumambient organs, are important adjunct to diagnosis. Immunohistochemistry is an essential mean to confirm diagnosis of mesothelioma. In summary, myxoid mesothelioma is one type of mesothelioma recapitulating the secretory function of the normal mesothelium. Further studies are needed to resolve the paradoxical problem about the survival.
